# The psychosocial impact of two non-destructive earthquakes on pupils in Cephalonia island: a comparison with the Athens earthquake of September 1999

**DOI:** 10.1080/20008198.2017.1351223

**Published:** 2017-09-29

**Authors:** Kalliopi Triantafyllou, Vasiliki Ntre, Gerasimos Kolaitis

**Affiliations:** ^a^ Department of Child Psychiatry, Medical School, National and Kapodistrian University of Athens, “Aghia Sophia” Children’s Hospital, Athens, Greece

**Keywords:** Earthquakes, psychosocial impact, post-traumatic stress disorder (PTSD), children and adolescents

## Abstract

**Background**: Natural disasters may have a serious impact on child and adolescent mental health. High rates of post-traumatic stress symptoms (PTSS), post-traumatic stress disorder (PTSD) and, to a lesser degree, associated psychopathology following natural disasters have been reported in many countries, including Greece. It has been suggested that whether or not youths will report PTSS/PTSD after a traumatic experience depends on different factors, such as the experienced threat to life, severity of exposure, pre-existing psychopathology, parental reactions to the traumatic experience, and adequacy of the care provided (e.g. Furr et al., 2010, Kiliç et al., 2003). Greece is a country where earthquakes occur frequently and sometimes may be destructive. On 7 September 1999, a disastrous earthquake occurred in Athens which was followed by 4000 aftershocks and resulted in 143 deaths, more than 400 injuries and damage to approximately 75,000 households. One hundred and fifteen schoolchildren who lived at the epicenter of the earthquake were assessed and were compared to 48 children not affected by the earthquake. There was a high rate (78%) of severe to mild PTSS in the earthquake-exposed group; 32% of these children scored above cut-off for depression symptoms compared to the control group (12.5%). Severe or moderate symptoms of PTSD were related to elevated scores of depression symptoms (Kolaitis et al., 2003). More recently, Cephalonia Island was affected by two earthquakes (26 January and 3 February 2014) with almost the same magnitude (6.0 Richter). One hundred and eighty-six houses in the area of the epicenter and 17 houses near the epicenter and far away from it suffered severe damage. No fatalities or serious injuries were reported. The aim of the current study was to examine PTSS and other psychosocial problems in youths who experienced the earthquake of Cephalonia and compare these results with those from the earthquake in Athens.

**Method**: 520 pupils aged 11–18 (mean age 14.26 years, 39.2% boys) who lived in three districts, i.e. in the epicenter, near the epicenter, and far away (according to the distance from the earthquake epicenter,) participated in the current study by completing questionnaires.

**Results**: Youths living in areas on or near the epicenter reported higher PTSS compared to those living far away from the epicenter (*p* < .01). Five percent of the adolescents were likely to meet diagnostic criteria for posttraumatic stress disorder (PTSD), without differences between the districts. No differences in anxiety and depression symptoms were found between the three groups of youths.

**Conclusions**: Comparing the Athens earthquake and those of Cephalonia Island, pupils from Cephalonia reported fewer symptoms compared to those in Athens. It seems that the magnitude of the disaster (not very destructive in Cephalonia, since houses and other buildings were reconstructed according to guidelines for earthquake-resistant construction, after the Great Cephalonia Earthquake of 1953), the severity of the traumatic experience and the local residents’ familiarity with earthquakes (since Cephalonia is one of the most earthquake-prone areas of Greece) play a significant role in youths’ mental health following a natural disaster.
